# Supplementary figures and images for: Quantum engineered MXene–graphene–plasmonic nanocomposites for next-generation transparent and flexible space photovoltaics
Source: Turk J Chem. 2025 Jan 1;50(3):243–58. doi: 10.55730/1300-0527.3795 (PMC13384565; doi:10.55730/1300-0527.3795)

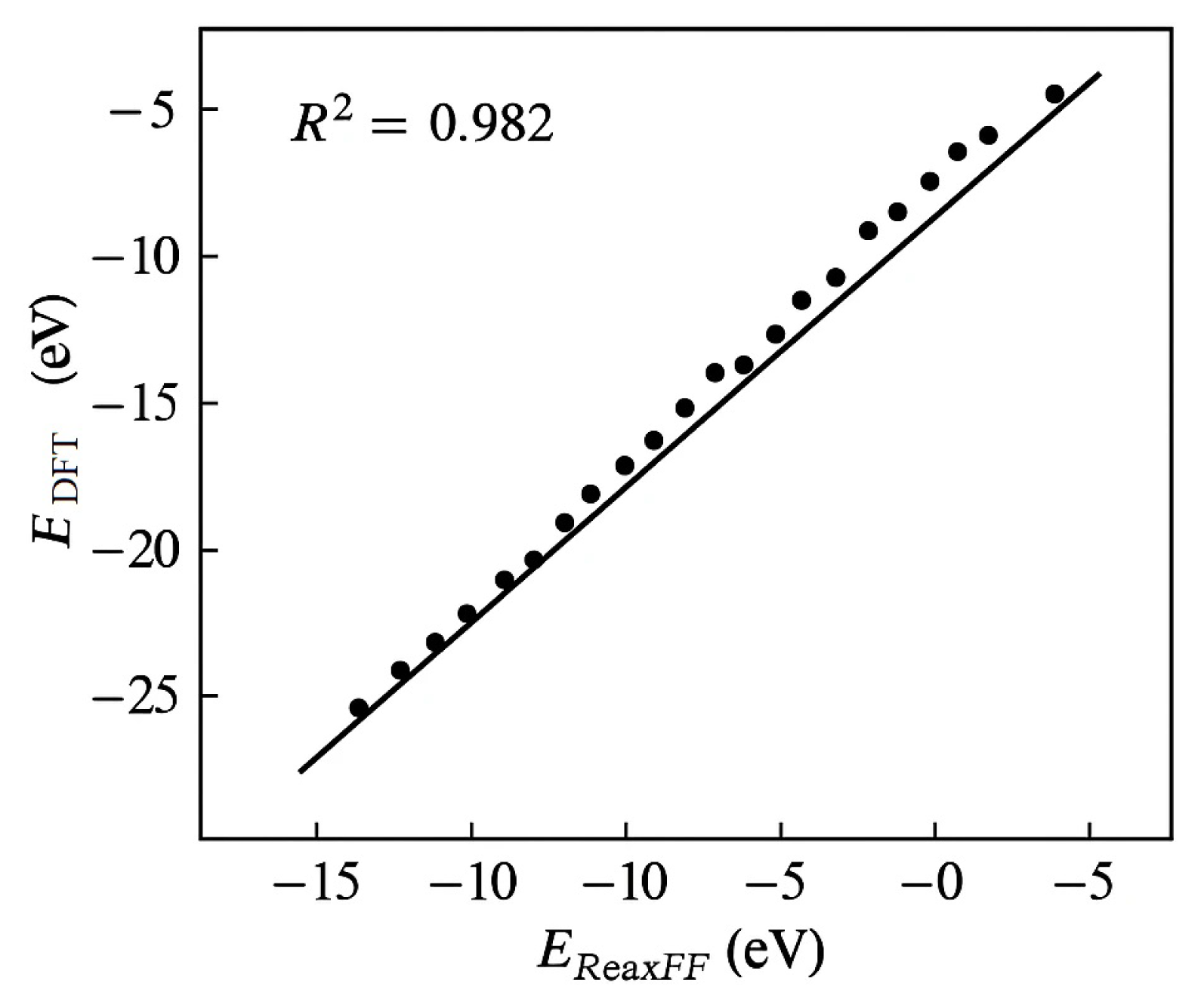

Supplement: Figure S1 — ReaxFF parameter fitting and validation. Correlation between ab initio (HSE06) and ReaxFF-predicted total energies for the Ti3C2Tx–Au–graphene hybrid system. The fitted dataset covers 135 configurations with energy range of ≈ 9 eV per atom. The excellent agreement (R2 = 0.982) confirms the reliability of the force-field parameterization used in molecular dynamics simulations. [file tjc-50-03-243s1.tif]

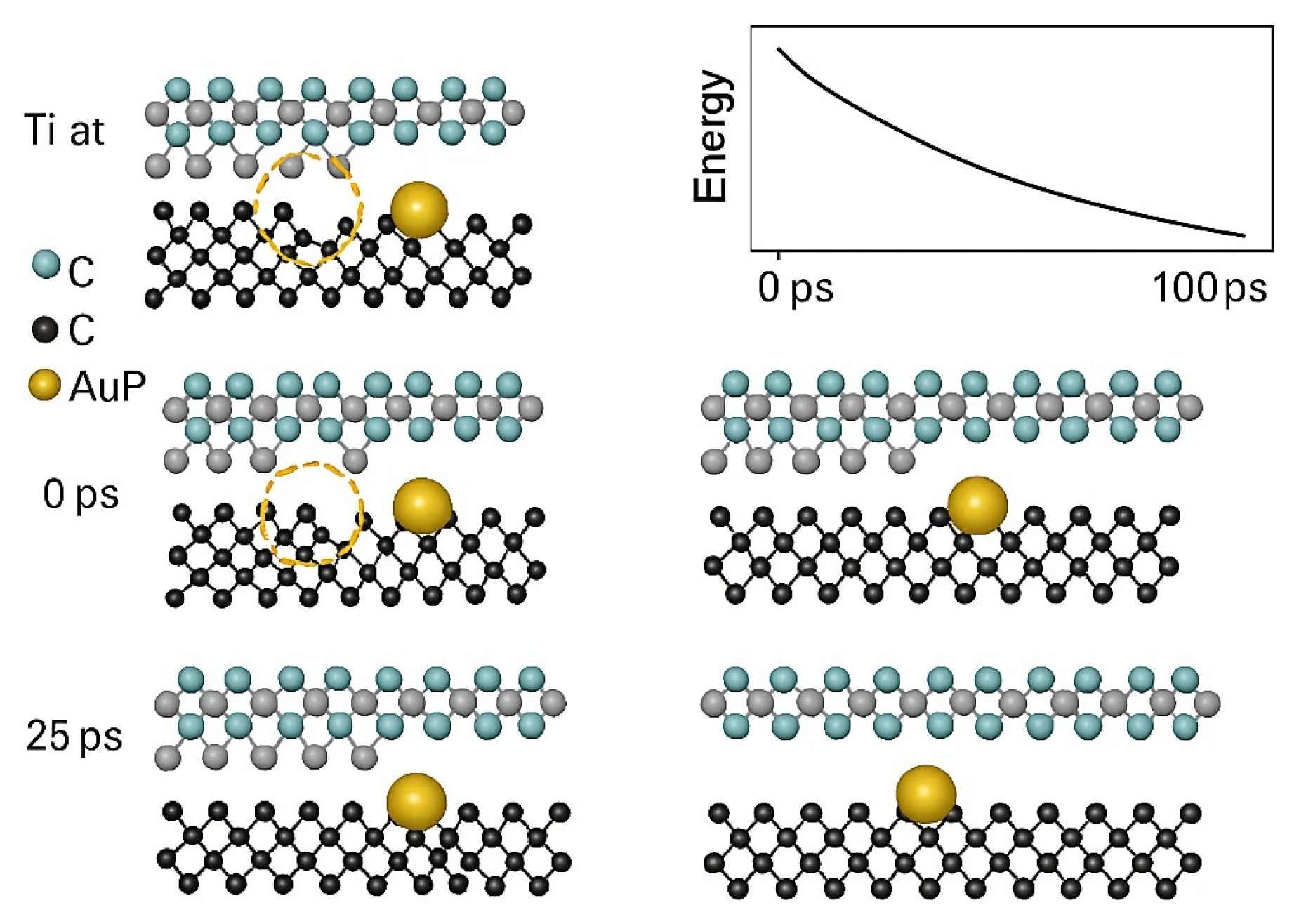

Supplement: Figure S2 — AIMD snapshots illustrating the self-healing process. Sequence of ab initio molecular dynamics configurations recorded at 0, 30, 60, and 100 ps during relaxation at 300 K. Defect recovery proceeds through atomic diffusion and recombination of Ti-vacancy pairs, quantified using the healing efficiency index ηh = 1 – (Edef / E0) ≈ 0.87. The system fully regains lattice continuity after ≈ 90 ps, indicating robust intrinsic self-repair capability. [file tjc-50-03-243s2.tif]

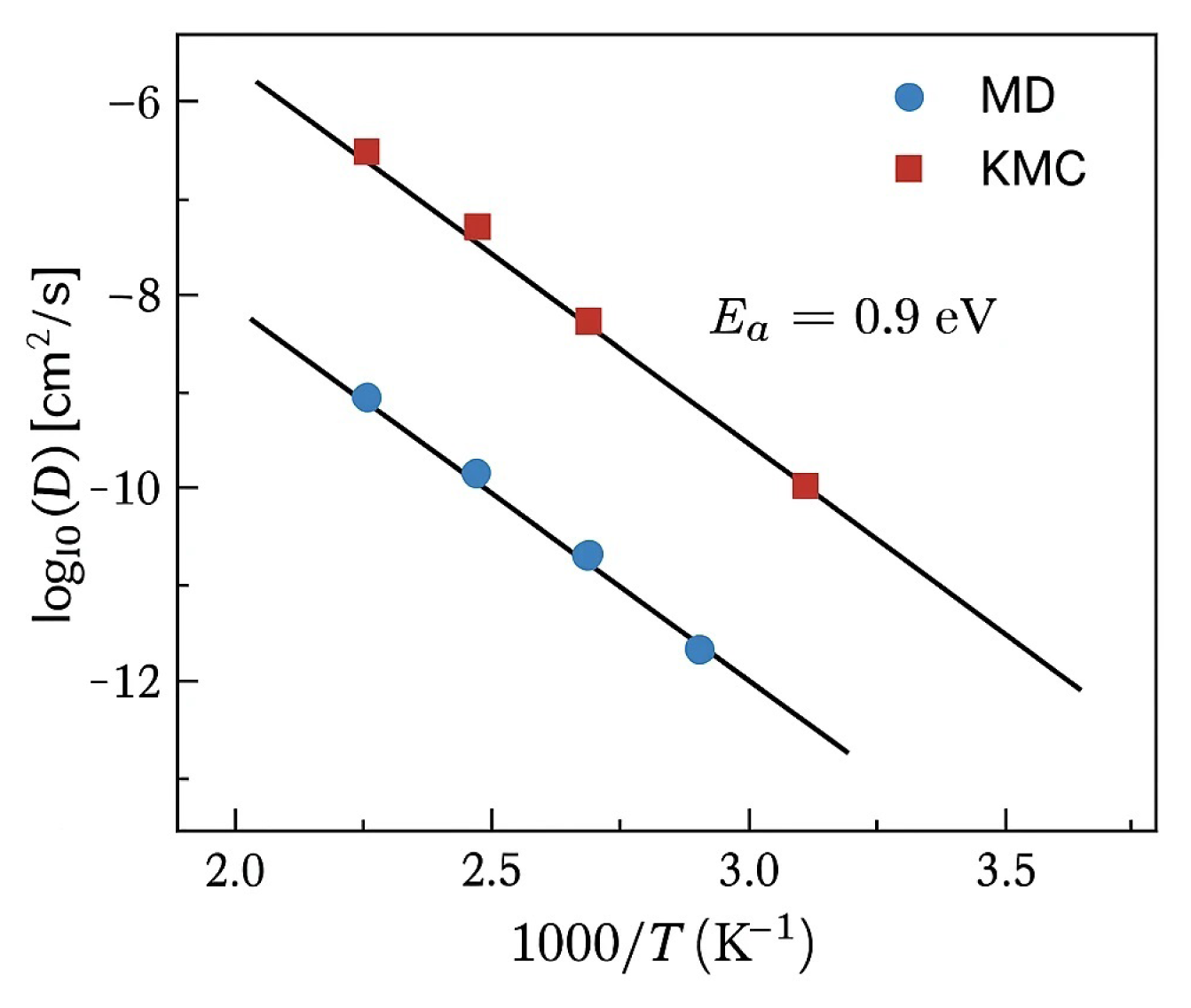

Supplement: Figure S3 — Defect diffusion validation across MD and KMC scales. Arrhenius plots comparing diffusion coefficients obtained from molecular dynamics (~500 atoms) and kinetic Monte Carlo (~106 sites) simulations. Both datasets exhibit consistent slopes with activation energy Ea ≈ 0.9 eV and extrapolated pre-exponential factor D0 ≈ 10−8 cm2/s, validating the cross-scale transferability of defect-migration kinetics. [file tjc-50-03-243s3.tif]
